# Bio-inspired feature selection for early diagnosis of Parkinson’s disease through optimization of deep 3D nested learning

**DOI:** 10.1038/s41598-024-74405-5

**Published:** 2024-10-08

**Authors:** S. Priyadharshini, K. Ramkumar, Subramaniyaswamy Vairavasundaram, K. Narasimhan, S. Venkatesh, P. Madhavasarma, Ketan Kotecha

**Affiliations:** 1 School of Electrical and Electronics Engineering, SASTRA Deemed University, Thanjavur, India; 2 School of Computing, SASTRA Deemed University, Thanjavur, India; 3 School of Computer Science and Engineering, Vellore Institute of Technology, Vellore, India; 4 Symbiosis Centre for Applied Artificial, Symbiosis Institute of Technology, Symbiosis International (Deemed University), Pune, India

**Keywords:** Deep nested 3D learning, Feature fusion, Feature optimization, Magnetic resonance imaging, Multi-classification, Parkinson’s diseases, Computational biology and bioinformatics, Diseases, Health care, Engineering

## Abstract

Parkinson’s disease (PD) is one of the most common neurodegenerative disorders that affect the quality of human life of millions of people throughout the world. The probability of getting affected by this disease increases with age, and it is common among the elderly population. Early detection can help in initiating medications at an earlier stage. It can significantly slow down the progression of this disease, assisting the patient to maintain a good quality of life for a more extended period. Magnetic resonance imaging (MRI)-based brain imaging is an area of active research that is used to diagnose PD disease early and to understand the key biomarkers. The prior research investigations using MRI data mainly focus on volume, structural, and morphological changes in the basal ganglia (BG) region for diagnosing PD. Recently, researchers have emphasized the significance of studying other areas of the human brain for a more comprehensive understanding of PD and also to analyze changes happening in brain tissue. Thus, to perform accurate diagnosis and treatment planning for early identification of PD, this work focuses on learning the onset of PD from images taken from whole-brain MRI using a novel 3D-convolutional neural network (3D-CNN) deep learning architecture. The conventional 3D-Resent deep learning model, after various hyper-parameter tuning and architectural changes, has achieved an accuracy of 90%. In this work, a novel 3D-CNN architecture was developed, and after several ablation studies, the model yielded results with an improved accuracy of 93.4%. Combining features from the 3D-CNN and 3D ResNet models using Canonical Correlation Analysis (CCA) resulted in 95% accuracy. For further enhancements of the model performance, feature fusion with optimization was employed, utilizing various optimization techniques. Whale optimization based on a biologically inspired approach was selected on the basis of a convergence diagram. The performance of this approach is compared to other methods and has given an accuracy of 97%. This work represents a critical advancement in improving PD diagnosis techniques and emphasizing the importance of deep nested 3D learning and bio-inspired feature selection.

## Introduction

Parkinson’s disease (PD) is the second most common neurological illness that affects older people. Clinicians typically observe various symptoms, which include motor and non-motor signs such as tremors, stiffness, bradykinesia (slow movement), and cognitive decline, to diagnose PD. Even though the research community has been exploring this domain for the past two decades, PD is still a disease with no direct clinical intervention-based cure. The traditional method of diagnosis is based on clinical data, neuroimaging data, and protein biomarkers, which are used to evaluate stages of PD. Magnetic Resonance Imaging (MRI) is a non-invasive medical imaging technique to identify PD that produces 3Dimensional (3D) anatomical images, which can be used to detect the changes in the structure of the brain and spot iron deposition in the Substantial Nigra (SN)^1^. Different anatomical and morphological structures of the brain are studied by using various relaxation sequences of MRI scanning techniques such as T1-weighted, T2-weighted, proton density and FLuid Attenuated Inversion Recovery (FLAIR) to provide deeper insights for further analysis. It is found that T2 MRI can detect iron accumulation changes in MR signals quite effectively, revealing alterations in iron content in PD patients. T2 weighted images are utilized to identify structural changes in the SN due to dopamine neuron loss associated with PD.

Recently, Medical image processing has undergone a revolution with the advent of Artificial intelligence (AI), specifically deep neural networks, resulting in better diagnostic accuracy than traditional AI approaches^2^. The AI research community have applied various Machine Learning (ML) techniques such as Support Vector Machine (SVM), Random Forest (RF), and Bayes Classifier to classify Control and PD persons using MRI and has obtained considerable improvement in accuracy in the past two decades. ML employs data pipeline methodologies such as Feature Extraction and Feature Selection. Recently, researchers have shown interest in using Voxel-Based Differences to extract features from control and PD individuals. Principal Component Analysis has been used to reduce the dimensions of the features for better classification. However, all these methods require a deeper level of understanding of domain knowledge to extract appropriate features for diagnosing PD. They also have limitations in scalability (making them work for larger datasets quite tricky), and they rely heavily on manually choosing the relevant features. To solve these problems, automatic feature detection, without human intervention, based on Deep Learning (DL) approaches, has become a viable solution for addressing the issues mentioned above.

DL algorithms have shown better performance, especially in multi-class classification problems. Convolutional Neural Networks (CNN), a class of DL algorithm, has achieved cutting-edge results in handling clinical data, which includes audio, video and image data for medical diagnosis applications^3,4^.The main challenge in medical imaging is that it is difficult to acquire patient data, so CNN, which operates with millions of parameters, may overfit with smaller data sets. This issue can be addressed by using transfer learning approaches to enhance the number of data points and facilitate CNN effectively learning from sparse data. The application of transfer learning dramatically enhances the performance of DL algorithms compared to traditional ML techniques^5^. In recent years, several pre-trained DL architectures like Residual Networks (ResNets), EfficientNets, and Vision Transformers (ViTs) have been used widely by the research community^6–10^. These architectures have proven quite effective in handling different modalities of data such as MRI, Single Photon Emission Computed Tomography (SPECT), Dopamine Transporter Scan (DaTscan) and other neuroimages for accurate diagnosis. This study compares the proposed framework with ViT and EfficientNet, which provides a rigorous benchmark to validate the proposed framework’s efficacy in PD detection.

AI has shown remarkable progress in healthcare, particularly in handling MRI data with interpretable features for effective diagnosis, aiding doctors’ decision-making process. However, a significant challenge arises from the uncertainty in the generalisation of these models in handling validation data. To address this, a novel method is proposed for detecting PD using a hybrid Deep Learning architecture. In the proposed framework, the 3D-CNN and 3D-ResNet models are individually analyzed for multi-class classification. Specifically, each model extracts features from the input data, providing distinct feature sets. The extracted features from the 3D-CNN and 3D-ResNet are then fused using Canonical Correlation Analysis (CCA) to integrate the complementary information from both models. After feature fusion, a feature selection process is applied using Whale Optimization Algorithm (WOA) to refine the feature set by selecting the most relevant features. Finally, various ML algorithms are applied to this selected feature set for multi-class classification. This approach ensures a comprehensive analysis by leveraging the strengths of both DL models and optimizing the feature set for improved classification performance. Through this proposed approach, a significant improvement is obtained in the early detection of PD compared to the state-of-the-art approaches in the literature.

The key contributions of this work are as follows:

1. Developed a strategic hyperparameter tuning technique based on a novel 3D-CNN model for automated feature extraction from MRI scans, improving accuracy and efficiency in PD diagnosis.
2. Conducted a comprehensive analysis of 3D-CNN, 3D-ResNet, Vision Transformer, and EfficientNet models, demonstrating that 3D-CNN and 3D-ResNet consistently outperformed others in accuracy and robustness for PD diagnosis.
3. Fused features from 3D-CNN and 3D-ResNet models using CCA, enhancing feature representation. Applied an Optimized feature selection approach using bio-inspired metaheuristics with a WOA, which resulted in improved performance.
4. Developed an intelligent system, integrating deep learning and machine learning methods for classifying PD using whole-brain imaging techniques. This approach leverages the concept of diverse feature integration techniques for improved classification accuracy.
5. Demonstrated significantly enhanced performance metrics and accuracy through the proposed fusion approach, validating its effectiveness in PD diagnosis.

The research article is presented as follows: section “Related works” highlights the contemporary state-of-the-art research work in PD diagnosis. In section “Materials and methods”, the results and the detailed steps involved in the work execution are explained. Section “Results” focuses on the analysis carried out on the performance of the proposed model in comparison with other models existing in the literature. Section “Discussion” concludes with a summary of the key achievements of the proposed work. An overview of the methodology, algorithmic techniques, and dataset used for this study is given in section “Conclusion”.

## Related works

In recent years, AI technologies have made significant progress and are being widely used in many healthcare industries. It is interesting to observe that the medical field has fully embraced this advancement in technologies and intense learning methods to diagnose diseases like Alzheimer’s and PD with multiple modalities of data. The researchers have deployed various advanced ML approaches to improve PD detection in the past two decades. In^11^ it is observed that the authors have come up with a novel way of integrating the entropy weight with the K-nearest neighbors (KNN) algorithm, resulting in improved accuracy compared to conventional methods. The performance of this algorithm is better when compared with RF, Naive Bayes, and vanilla KNN algorithms. In^12^a novel disease prediction method is developed by preprocessing MRI scans and subsequently classified using Recurrent Neural Network (RNN) architecture. This approach involves extracting attributes from specific brain regions, such as the caudate and nuclei, demonstrating the potential of deep learning techniques in medical image analysis.

A method for differentiating Parkinson’s Disease (PD) from healthy controls (HC) utilizing neuromelanin-sensitive MRI (NMS-MRI) slices has been developed, where a 2D Convolutional Neural Network (2D-CNN) is implemented to identify prognostic and diagnostic biomarkers within the region of interest in brain morphological structures^13^. In^14^ the authors have proposed a method for identifying the subcortical region, SN, as the Region of Interest (ROI) in DL-based PD classification. By leveraging CNN and other architectures, this study highlights the importance of accurate feature selection in improving disease detection accuracy. In^15^ Parkinson’s Progression Markers Initiative (PPMI) database is utilized to evaluate the disease classification accuracy of a 3D ResNet model. This study achieves higher accuracy rates through a 5-fold cross-validation strategy and also employs an explainability framework to analyze feature map biases and generate visual representations. Further, saliency maps are generated in this work to provide explainable artificial intelligence, highlighting the brain areas that contribute most to the classification task.

Further, a deep learning model incorporating various transfer learning methods is proposed to accurately distinguish between PD patients and HC participants. The model is trained on a large and diverse database, integrating deformation fields and essential clinical features, which contributes to its robustness and accuracy in PD detection^16^. Moreover, a CNN-based approach has been applied to classify PD and non-PD subjects using MRI data from various datasets, achieving an accuracy of 79.3%. This method underscores the potential of CNNs in medical imaging tasks^17^. In^18^ authors have proposed the use of a 3D-CNN model for disease detection using functional Magnetic Resonance Imaging (fMRI) data. The pre-processing involves Independent Component Analysis (ICA) and dual regression methods through FMRIB Software Library (FSL) to identify resting-state networks (RSNs) while eliminating noise and artifacts. The 3D-CNN model is trained using a 10-fold cross-validation technique with the identified RSNs or 3D spatial maps. In^19^, the authors suggest an innovative hybrid technique for accurately classifying brain tumors through multiple approaches. This strategy combines machine learning methods for feature classification with a unique CNN model for feature extraction. The study compares CNN performance using nine CNN models and optimizes hyperparameter values for ML classifier algorithms.

The literature survey highlights the importance of hybrid approaches in fusing ML and DL techniques in disease detection and classification. It also emphasizes the methodologies and contributions of each study, including feature fusion from images and meta-heuristic-based optimization processes, which play a significant role in achieving high accuracy. An overview of the literature survey is shown in Table 1. It summarizes the recent studies focusing on the classification of brain MRI scans using various deep-learning methods.

**Table 1:** Recent studies for the classification of brain MRI scans.

References	Problem	Method	Dataset	Accuracy (%)
^12^	Binary-classification	CNN-RNN	MRI and DaTscan data	98.0
^13^	Binary-classification	2D-CNN and CAM	MRI data	80
^15^	Binary classification	3D-ResNet	PPMI	94.5
^17^	Multi-classification	2D-CNN	PPMI	79.3
^18^	Multi-classification	3D-CNN	Tau Wu	86.0
^19^	Multi-classification	Hybrid model (pre-trained) + 2D-CNN	MRI data	97.1

## Materials and methods

**Figure 1:**
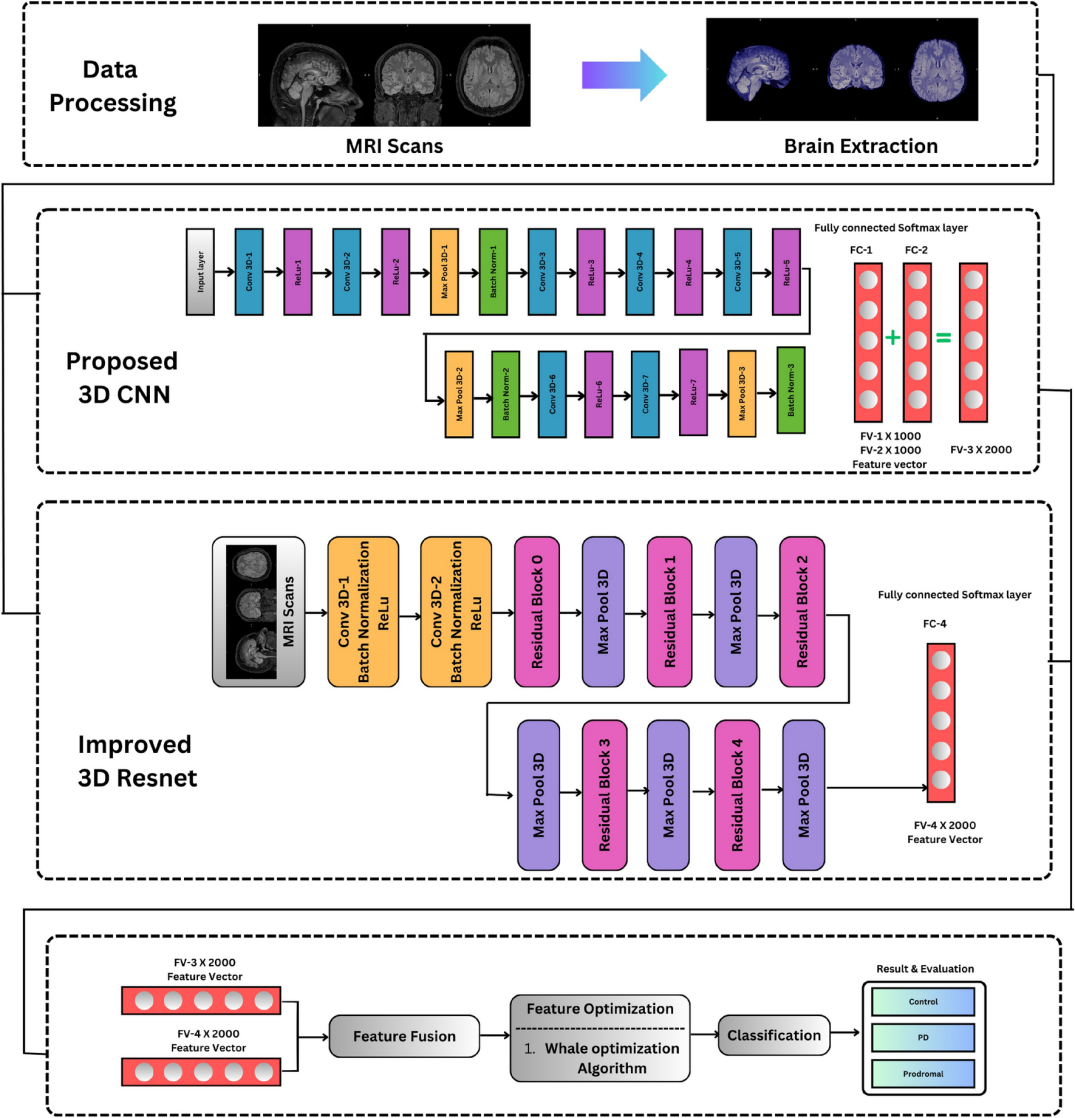
Overall architecture of the proposed method.

This section outlines the methodology and the algorithmic tools used in this proposed work with a focus on the collection of datasets, the development of different deep neural network architectures, and algorithmic changes carried out in realizing fusion and optimization techniques. The overall proposed methodology is illustrated in the block diagram in Fig. 1, which depicts the following modules, namely: (1) Proposed 3D- CNN architecture, (2) Improved ResNet model, (3) Canonical correlation-based fusion followed by whale optimization. Each of the modules is discussed in detail in this section.

### Data collection and pre-processing

The dataset used in the present work is collected from PPMI database^20^ ,which is an open-source repository with a collection of different modality images of control and PD subjects. In the proposed experimental investigation, a total of 303 MRI images, consisting of 110 control, 58 prodromal and 135 PD MRI images, were used. The analysis accounted for age and sex in MRI image quality, demonstrating the model’s effective management of demographic variability. From the literature, it is identified that T2-weighted MRI data is extensively used for localizing the SN region. This study performed the preprocessing steps like brain extraction, registration, normalization and data augmentation as described in the reference^21,22^. In^23^ author used to analyze the structural changes in subcortical regions to diagnose PD. Hence, among T1, T2 and FLAIR weighted MRI sequences, only the T2 weighted sequence is considered for the proposed method. To ensure the adaptability of the proposed framework within the current healthcare system, the output format and resolution of MRI scans from various MRI machines were analyzed and included in the training and testing data. The dataset includes MRI scans from 1.5T and 3T machines, with 3T images generally providing higher spatial resolution and signal-to-noise ratio. This ensures that the proposed classification model maintains its accuracy regardless of these factors. Significant factors of various MRI machines are listed in Table 2.

**Table 2:** A comparative overview of performance and image quality for different MRI machines.

MRI machine	Manufacturer	Magnetic field strength (T)	Output format	DICOM image quality factors
MAGNETOM Skyra	Siemens Healthineers	3	DICOM	High spatial resolution, high SNR, good artifact reduction
MAGNETOM Aera	Siemens Healthineers	1.5	DICOM	Moderate spatial resolution, good SNR, artifact management
SIGNA Premier	GE Healthcare	3	DICOM	High spatial resolution, excellent SNR, advanced sequences
Discovery MR750	GE Healthcare	3	DICOM	High resolution, high SNR, effective artifact reduction
Ingenia Elition	Philips Healthcare	3	DICOM	High spatial resolution, high SNR, noise reduction features
Achieva	Philips Healthcare	1.5	DICOM	Moderate resolution, good SNR, enhanced contrast sequences

### 3D-CNN

**Figure 2:**
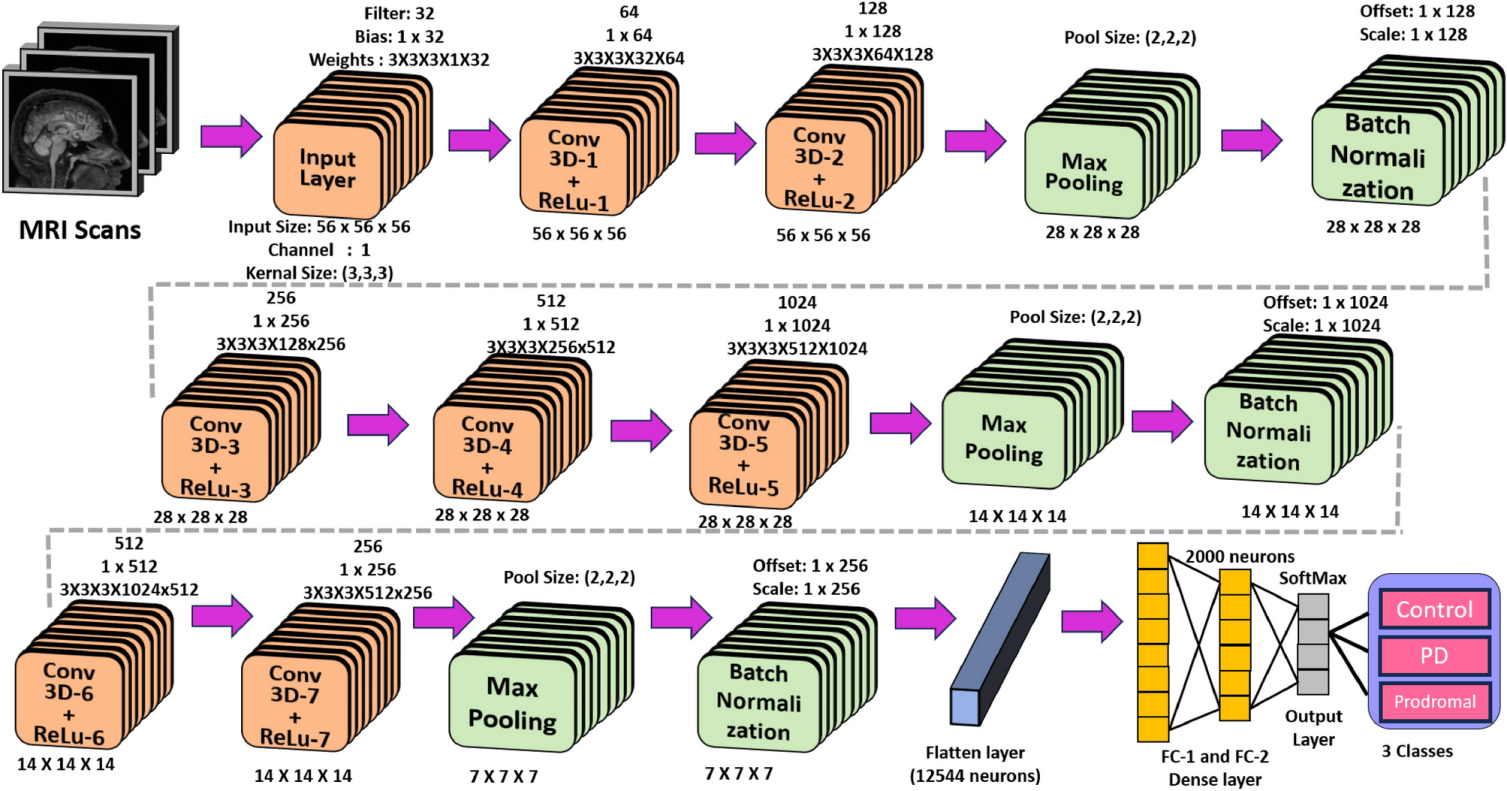
Proposed 3D-CNN.

A 3D-CNN architecture is designed and analyzed to classify input MRI images into 3 classes: control-0, prodromal-1, and PD-2. In the literature, 2D-CNN has been extensively explored^6,12–14^ for MRI data on 2D-slice classification. Since each subject has a variable number of slices in MRI scans, it is difficult to fix the input size of the architecture, which results in limited accuracy. In 3D-CNN architecture, three-dimensional MRI images can be directly given as input in the file format of the NIfTI. A novel 3D-CNN architecture is designed and constructed after performing extensive ablation studies. The proposed architecture comprises a total of twenty-four layers, which include an input layer, a 3D 3D conv layer with 3D Rectified Linear Unit (ReLU) activation functions, BN, a pooling layer, two dense layers, and a finally SoftMax classification layer. Figure 2, illustrates the architectural representation of the proposed 3D-CNN model.

### Improved 3D-ResNet

**Figure 3:**
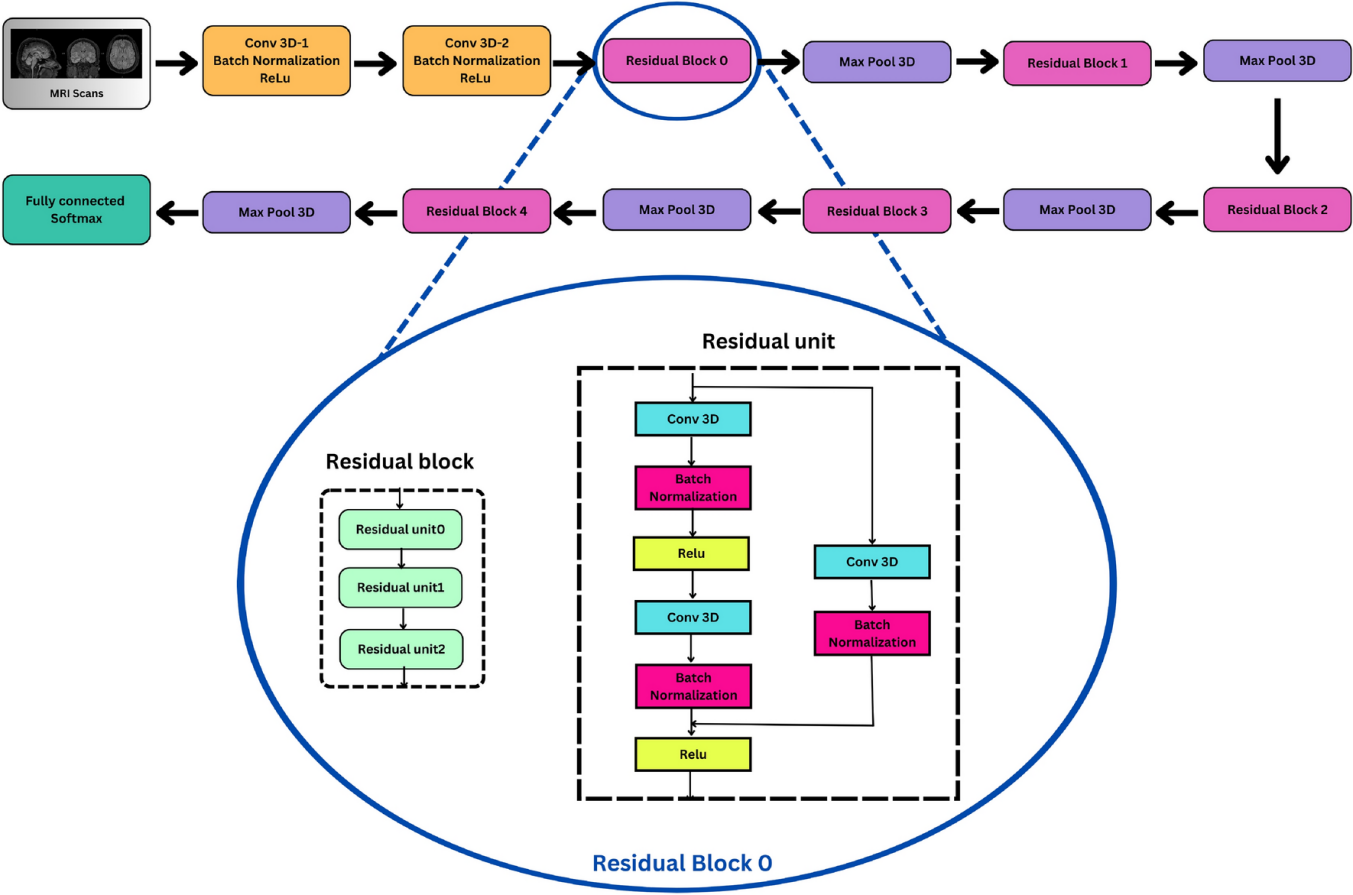
Improved 3D-ResNet.

In the literature, the Improved Resnet model^15^ is used to analyze and classify MRI images. In the proposed work, this model is used as one module for feature extraction. Further, the performance of the model is analyzed to classify MRI data. Figure 3 illustrates the architecture of the 3D-improved resnet model. This neural network architecture is composed of 15 layers, including an input layer, two conv3D blocks, five residual blocks with max-pooling layers, a fully connected layer, and a SoftMax layer. Each “conv3D block” consists of a 3D conv layer, BN, and ReLU activation layers. The residual block contains three residual units, each comprised of two 3D Conv layers, BN, and ReLU activation layers. A vital feature of the residual units is the skip connection, which directly adds the input to the output of the last ReLU layer. This study employs Adam optimization and the SoftMax classifier with a categorical cross-entropy loss function for the output layer optimization. The ReLU activation function, which is a non-linear function, is used to address the problem of vanishing or exploding gradients during training. The ReLU activation function can be defined as in Eq. (1):

1 $$\begin{aligned} f(y) = {\left\{ \begin{array}{ll} 0 & \text {if } y < 0 \\ y & \text {if } y \ge 0 \end{array}\right. } \end{aligned}$$

### Feature extraction

Feature extraction involves identifying relevant patterns from input image data. In a 3D CNN, the 21st and 22nd layers consist of fully connected layers, from these layers, 1000 features were extracted. For 23rd layer, feature concatenation is performed using algorithmic improvisations, which results in merging two distinct sets of features into one vector by selecting the highest values. Additionally, 1000 features are extracted from the 14th layer of an improved 3D ResNet. A total of 2000 features are finally extracted from both architectures.

### Feature fusion

Feature fusion is the process of combining or integrating features extracted from multiple deep neural network architectures (i.e. 3D-CNN and 3D-ResNet) to improve the performance or effectiveness of a machine learning model. In this work, feature fusion is performed using the CCA algorithm.

#### Feature concatenation

Feature concatenation involves merging two distinct sets of features into one vector by selecting the highest value^24^, as mentioned in Eq. (2). Concatenation of the features from the 21st and 22nd layer in the constructed 3D-CNN gives improved accuracy in PD diagnosis. The composite vector, denoted as $\tilde{x}_3$, is obtained by extracting the highest value from vectors $x_1 \in FV1$ and $x_2 \in FV2$. When $x_1$ and $x_2$ are equal, vector $\tilde{x}_3$ chooses one of these elements,and thereby avoiding duplication of elements. The pseudo-code for this process is outlined in Algorithm 1. Feature vector $\tilde{x}_3$ is with the dimensions $FV1 \times 1000$ and $FV2 \times 1000$. The concatenated feature map is expressed as follows:

2 $$\begin{aligned} \tilde{x}_3 = x_3 \leftarrow \max (x_1, x_2) \quad \text {and} \quad x_3 \in \text {Not Repeated} \quad \end{aligned}$$

**Algorithm 1:**
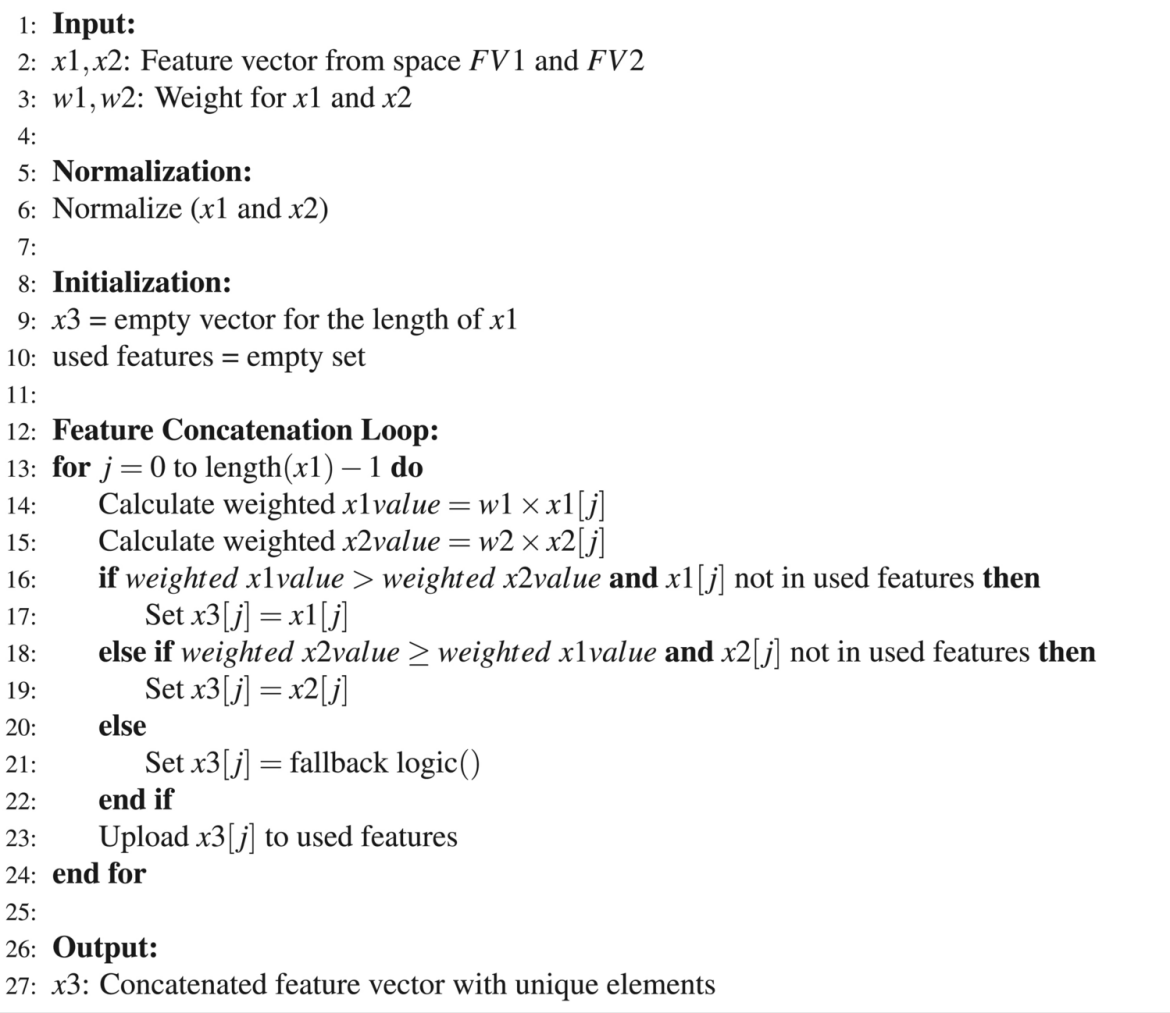
Pseudo-code for feature concatenation

#### Canonical correlation analysis

In this study, CCA is used to analyze correlations between two sets of features^25,26^. A total of 2000 features were extracted from both the 3D Resent and the 3D CNN modules. CCA is used to perform correlation-based analysis in this study. It is used to analyze associations between two sets of variables. Suppose $A \in \mathbb {R}^{p \times n}$ and $B \in \mathbb {R}^{q \times n}$ are two matrices, each contains $n$ training feature vectors from two different architectures. In other words, there are $n$ samples for each of which $(p+q)$ features have been extracted.

Let $S_{AA} = \mathbb {R}^{p \times p}$ and $S_{BB} = \mathbb {R}^{q \times q}$, it represents the set covariance matrices of $A$ and $B$, and $S_{AB} = \mathbb {R}^{p \times q}$ denote the between-set covariance matrix, where $S_{BA} = S_{AB}^T$. The comprehensive $(p+q) \times (p+q)$ covariance matrix, denoted as $S$, encapsulates all information regarding associations between pairs of features as shown in Eq. (3).

3 $$\begin{aligned} S = \begin{bmatrix} \text {cov}(A) & \text {cov}(A, B) \\ \text {cov}(B, A) & \text {cov}(B) \end{bmatrix} = \begin{bmatrix} S_{AA} & S_{AB} \\ S_{BA} & S_{BB} \end{bmatrix} \end{aligned}$$

This study employs CCA for feature fusion as it is designed to compute linear combinations optimally, denoted as $A^* = W_A^T A$ and $B^* = W_B^T B$, which results in maximizing the pairwise correlations across the two datasets as depicted in Eq. (4).

4 $$\begin{aligned} \text {corr}(A^*, B^*) = \frac{\text {cov}(A^*, B^*)}{\sqrt{\text {var}(A^*) \cdot \text {var}(B^*)}} \end{aligned}$$

where $\text {cov}(A^*, B^*) = W_A^T S_{AB} W_B$, $\text {var}(A^*) = W_A^T S_{AA} W_A$ and $\text {var}(B^*) = W_B^T S_{BB} W_B$. The maximization involves Lagrange multipliers, aiming to maximize the covariance between $A^*$ and $B^*$ while adhering to the constraints $\text {var}(A^*) = \text {var}(B^*) = 1$. The transformation matrices $W_A$ and $W_B$ are represented using Eq. (5),

5 $$\begin{aligned} \begin{aligned} S_{AA}^{-1} S_{AB} S_{BB}^{-1} S_{BA} \hat{w}_A&= \Lambda ^2 \hat{w}_A, \\ S_{BB}^{-1} S_{BA} S_{AA}^{-1} S_{AB} \hat{w}_B&= \Lambda ^2 \hat{w}_B. \end{aligned} \end{aligned}$$

where the diagonal matrix of eigenvalues, or squares of the canonical correlations, is denoted by $\Lambda ^2$ and the eigenvectors are represented by $\hat{w}_A$ and $\hat{w}_B$. The computed eigenvectors for the non-zero eigenvalues form the transformation matrices. The modified feature vectors are concatenated or summed up, to perform feature-level fusion to obtain the fused vectors $Z$. Here $Z_1$ and $Z_2$ are the Canonical Correlation Discriminant Features (CCDFs) mentioned in Eqs. (6, 7). The fused vector ($Z$) is applied to the optimization-based feature elimination module to refine and select important features that contribute significantly to the model’s predictive accuracy.

6 $$\begin{aligned} & Z_1 = (A^* \mid B^*) = \left( (W_A^T A) \mid (W_B^T B)\right) = \begin{bmatrix} W_A & 0 \\ 0 & W_B \end{bmatrix}^T \begin{pmatrix} A \\ B \end{pmatrix} \end{aligned}$$

7 $$\begin{aligned} & Z_2 = A^* + B^* = W_A^T A + W_B^T B = \begin{pmatrix} W_A \\ W_B \end{pmatrix}^T \begin{pmatrix} A \\ B \end{pmatrix} \end{aligned}$$

### Feature optimization

The primary goal of feature optimization is to identify the significant features responsible for predictions while also lowering the computational load of the system and data handling. The reduced feature dimension, after optimization, streamlines data management and facilitates easier handling of the training and prediction processes for larger datasets.

#### Mathematical model of WOA

Mirjalili and Lewis developed the Whale Optimization Algorithm (WOA) inspired by the humpback whales’ bubble-net feeding strategy^27,28^. This method, where whales trap their prey using bubbles in a circular pattern, is adapted in WOA to optimize the search for solutions to complex optimization problems.

**Encircling Prey:** In their hunting strategy, humpback whales locate and encircle their prey. This behavior is reflected in WOA, which assumes that the best current solution approximates the optimal solution^29^. Once the best search agent is identified, other agents converge towards it, mimicking the whales’ encircling behavior. This process is mathematically represented in Eqs. (8,9). Here, $\vec {Z}(u)$ represents the agent’s position, *u* represents the iteration and $\left( \vec {Z}^* \right)$ represents the optimal solution. In Eqs. (10, 11) $\vec {A}$ and $\vec {D}$ indicate convergence values. The random number is $\vec {s}[0,1]$, and $\vec {s}$ stands for the vector that decreases linearly from 2 to 0 during an iteration.

8 $$\begin{aligned} & \vec {Z}(u+1) = \left| \vec {Z}^*(u) - \vec {A} \cdot \vec {F} \right| , \end{aligned}$$

9 $$\begin{aligned} & \vec {F} = \left| \vec {D} \cdot (\vec {Z}^*(u) - \vec {Z}(u)) \right| , \end{aligned}$$

10 $$\begin{aligned} & \vec {A} = 2 \cdot \vec {a} \cdot \vec {s} - \vec {a}, \end{aligned}$$

11 $$\begin{aligned} & \vec {D} = 2 \cdot \vec {s} \end{aligned}$$

The **bubble-net attacking** method, employed by humpback whales, involves shrinking and spiraling towards the prey to tighten the search area. In the WOA, this is simulated by decreasing the value of $\vec {a}$ (see Eq. 11), which narrows the search space and mimics prey-catching behavior. As $\vec {A}$ is dependent on $\vec {a}$, it too linearly decreases from 2 to zero. The spiral movement pattern of the whales, which is crucial for capturing their prey, is mathematically modelled in the equations in (12, 13).

12 $$\begin{aligned} & \vec {F}^* = \left| \vec {Z}^*(u) - \vec {Z}(u)\right| , \end{aligned}$$

13 $$\begin{aligned} & \vec {Z}(u+1) = e^{bk} \cos (2\pi k) \vec {F}^* + \vec {Z}^*(u) \end{aligned}$$

The distance between the whale and its target prey, denoted as $\vec {F}^*$. The parameter *b* is the logarithmic spiral constant, and *l* is a random value between $[-1, 1]$. Humpback whales exhibit a 50% likelihood of choosing between a spiral or a direct narrowing approach toward their prey.The parameter determines the probability of selecting either movement pattern *p* in Eq. (14), a random value between $[0, 1]$.

14 $$\begin{aligned} \vec {Z}(u+1) = {\left\{ \begin{array}{ll} \vec {Z}^* - \vec {A} \cdot \vec {F}, & \text {if } p < 0.5 \\ e^{bk} \cos (2\pi k) \vec {F}^* + \vec {Z}^*(u), & \text {if } p \ge 0.5 \end{array}\right. } \end{aligned}$$

In the **exploration phase** of WOA, $\vec {A}$ is randomly assigned a value between $[-1,1]$, prompting the search agents to diverge from the reference whale. This promotes a global search as the updated position of a search agent is determined by randomly selecting another agent. The mathematical formulation of this exploration mechanism is outlined in Eqs. (15, 16), where $\vec {Z}_{\text {rand}}$ is a random location for a random whale chosen from the current population.

15 $$\begin{aligned} & \vec {Z}(u+1) = \vec {Z}_{\text {rand}} - \vec {A} \cdot \vec {F}, \end{aligned}$$

16 $$\begin{aligned} & \vec {F} = |\vec {C} \cdot (\vec {Z}_{\text {rand}} - \vec {Z})| \end{aligned}$$

#### WOA for feature selection

This study seeks to enhance feature selection for PD classification using the WOA, inspired by bio-inspired algorithms. Extracted 2000 features from the FC-3 layer of a 3D-CNN model and the FC-4 layer of a 3D ResNet model. Effective feature selection is crucial for improving data representation and boosting performance based on specific criteria. Optimal feature selection not only simplifies the model but also enhances the estimator’s generalisation ability, learning speed, and overall performance. Common issues include stagnation at local optima and high computational costs. Thus, it is imperative to employ efficient global search strategies to tackle the feature selection problem. In WOA, each feature subset is analogous to the position of a whale within the search space. Each subset may include up to $N$ corresponding to the number in the original dataset. The quality of each solution is judged based on two criteria: the number of features and its classification accuracy; the fewer the features and the higher the accuracy, the better the solution. Solutions are evaluated using a fitness function that combines these two objectives: the accuracy achieved using a K-Nearest Neighbors (KNN) classifier and the number of features selected.

Solution Representation is a critical challenge in designing metaheuristic algorithms. In this study, we represent solutions as one-dimensional vectors with $N$ elements, where $N$ matches the features in the dataset. Each element in the vector is binary, marked as “1” or “0.” A “1” indicates the selection of the corresponding feature, while a “0” denotes its exclusion. This binary approach efficiently delineates the inclusion and exclusion of features in the solution set. The fitness function in our proposed method is carefully crafted to balance the number of selected features (minimized) against the classification accuracy (maximized). The function is defined as in Eq. (17):

17 $$\begin{aligned} f_\theta = \alpha \gamma _R (D) + (1-\alpha ) \frac{|R|}{|N|} \end{aligned}$$

**Figure 4:**
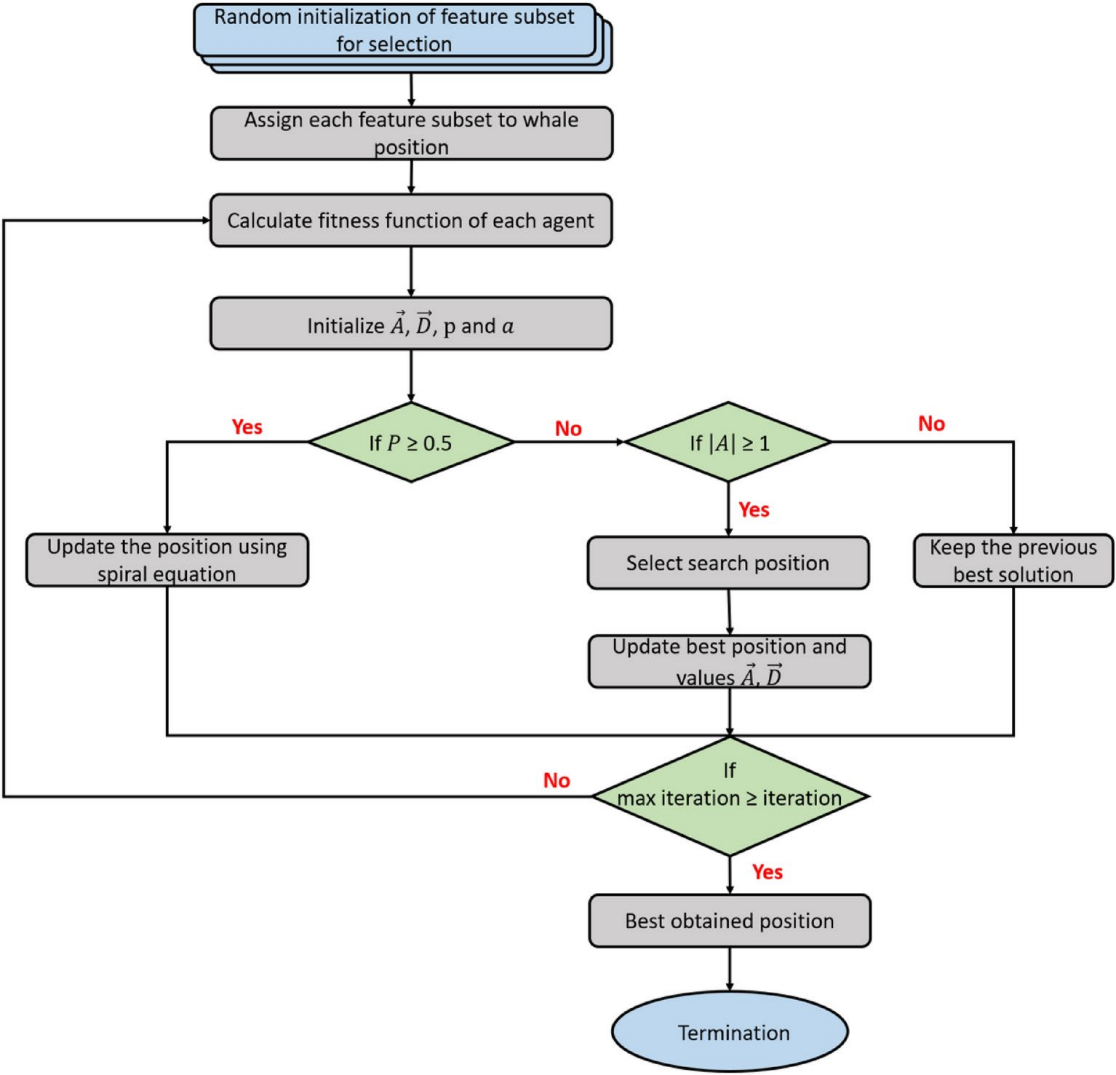
Feature selection process using WOA.

Here, $f_\theta$ is the fitness function, $\gamma _R (D)$ represents the classification error rate using KNN classifier. $|R|$ is the cardinality of the selected subset and $|N|$ is the total number of features in the dataset. The parameter $\alpha$ balances the importance of classification accuracy, while $\beta = (1 - \alpha )$ adjusts the weight given to the subset size. The values for $\alpha$ and $\beta$ are derived to reflect the trade-off between these two aspects, ensuring an effective evaluation of potential solutions^30^. The optimization process begins by initializing the coefficient vectors $\vec {A}$ and $\vec {D}$, and setting the initial best solution vector $\vec {Z}^*$ to a randomly chosen solution. The process continues through a predefined number of iterations, during which each whale’s position is updated according to Eqs. (8, 9). The fitness of each position is evaluated using the designated fitness function. If a better solution is identified during any iteration, the best solution vector $\vec {Z}^*$ is updated accordingly. This optimization cycle is repeated for a specified number of independent runs. The process terminates once the designated number of iterations is reached, concluding the search for the optimal solution. The detailed flow representation of WOA for feature selection is shown in Fig. 4.

## Results

### Model-1 (3D-CNN) architecture analysis

A total of 303 MRI images in the format of Digital Imaging and Communication in Medicine (DICOM), belonging to three different classes, are collected from the PPMI dataset repository. These DICOM images, each comprising a varying number of slices in the range of 100-200 images for a single subject. The collected DICOM images of each subject are converted to a single 3D Neuroimaging Informatics Technology Initiative (NifTi) format file. The NifTi file is further subjected to pre-processing, which includes skull stripping and field correction, with the objective of enhancing MRI data quality^31^. Finally, the input size is converted to 56x56x56 for subsequent analysis.

#### Ablation study

The study initially analyzed a basic 3D-CNN architecture, as shown in Table 3. The ablation study focuses on selecting the optimal number of convolution and max pooling layers to achieve desirable performance in terms of targeted accuracy. The study was carried out in multiple stages. In stage 1, the base model was designed with 8 layers, starting with the input layer, followed by two 3D- convolutional (3D-conv) layers, an activation layer, a pooling layer, a Batch Normalization (BN) layer, a flattened layer, and a fully connected SoftMax layer, which resulted in a model accuracy of 82.02%. In stage 2, a slightly modified version of the original base model, variant 1 architecture was designed with a 9-layer architecture, with an input layer, three 3D-conv layers, an activation layer, a pooling layer, BN layer, a flattened dense layer, and a SoftMax layer, achieving a model accuracy of 85.75%. In stage -3, Variant 2 was designed with a 17-layer architecture, including an input layer, two 3D-conv layers, activation layers, a pooling layer, a BN layer, three 3D-conv layers, activation layers, a pooling layer, a BN layer, a flatten dense layer, and a SoftMax layer, achieving an accuracy of 88.76%. Variant 3 comprises a 24-layer architecture, starting with an input layer, two 3D-conv layers, activation layers, a pooling layer, a BN layer, three 3D-conv layers, activation layers, a pooling layer, BN layer, two 3D-conv layers, activation layers, a pooling layer, a BN layer, and finally a flatten dense layer followed by a SoftMax layer, achieving the highest accuracy of 89.43%.

**Table 3:** Ablation study of the constructed 3D-CNN architecture.

Study 1: Modify the convolutional layer and max pool layer				
Model	No. of Conv_layer	No. of pooling layer	Test accuracy (in %)	Finding
Base model	2	1	82.02	Lowest accuracy
Variant 1	3	1	85.75	Intermediate
Variant 2	2+3	1+1	88.76	Intermediate
Variant 3	2+3+2	1+1+1	89.43	Highest

Study 2: Modifying the different parameters for four approaches				
Parameters	Base model	Variant 1	Variant 2	Variant 3
Pooling layer				
1. Max	$\checkmark$		$\checkmark$	
2. Average		$\checkmark$		$\checkmark$
Activation function				
1. ReLu	$\checkmark$	$\checkmark$	$\checkmark$	$\checkmark$
2. PreLu				
Batch size				
1. 32				$\checkmark$
2. 64	$\checkmark$	$\checkmark$	$\checkmark$	
Flatten layer				
1. Flatten	$\checkmark$		$\checkmark$	
2. Global max		$\checkmark$		$\checkmark$
Optimizer				
1. Adam	$\checkmark$	$\checkmark$	$\checkmark$	$\checkmark$
2. SGD				
Learning rate				
1. 0.01	$\checkmark$		$\checkmark$	
2. 0.001		$\checkmark$		$\checkmark$
Epoch				
Epoch	30	30	30	30
Evaluation metrics				
Accuracy	84.96	88.25	90.28	93.41
Precision	87.34	89.93	91.83	95.76
Recall	81.54	84.28	87.72	88.98
F1-score	85.32	87.43	90.81	91.68

Based on the selection of optimal architectures, with a base model and its three variants, further studies were carried out in this study with a focus on the appropriate selection of hyperparameters with exhaustive simulations. The hyperparameters were selected based on different criteria as listed in Table 3 under section study 2. This study aims to arrive at an optimal configuration for maximizing accuracy through changes in hyperparameters. This involves experimenting with various combinations of parameters across each model (Base model, Variant 1, Variant 2 and Variant 3). These parameters include the type of pooling layer, activation function, batch size, flattening layer method, optimizer, and learning rate. Out of all these approaches, variant 3 performs better than the other models in terms of various evaluation criteria. The Ablation study has proven to be very effective, as it improved the classification capabilities of the proposed model from 89.43 to 93.41%, showing a significant improvement of 3.98%.

#### Performance comparison of feature extraction layers in 3D-CNN for PD Prediction

In the field of medical diagnostics, the evaluation criteria involve analyzing four primary classification performance metrics: recall (Sensitivity/true positive rate/TPR), accuracy, precision (positive predictive value/PPV), and F1-score. Analysis 1 focuses on features extracted from the FC-1 (21st layer of the 3D-CNN, and various ML methods were applied and explored to achieve better classification results. Analysis 1, carried out with various ML models and their performance, as shown in Table 4, reveals that the Random Forest (RF) model achieved the highest accuracy at 88.7%. Analysis 2 examined the extracted features from the FC-2 (22nd) layer of the 3D-CNN and further processed using various ML classifiers. It is observed that the Gradient Boosting (GB) classifier achieved 90.1% accuracy, better than the other classifiers employed in this analysis. Analysis 3 combined features from both FC-1 and FC-2 layers (referred to as FC-3), and the extracted features were further processed using various ML methods for classification. This fusion of features resulted in increased accuracy, with GB achieving a much better performance when compared to the other classifiers, with a superior accuracy of 93.5%. In this analysis, metrics such as accuracy, precision, recall, and F1-score were used to evaluate the performance of the classifiers. These findings highlight the importance of selecting appropriate feature extraction layers for improving PD prediction accuracy.

**Table 4:** Comparative analysis of PD prediction performance using different architectural models and feature extraction methods.

Methods	Accuracy	Recall	Precision	F1-score
Analysis 1: PD prediction using extracted features from 3D-CNN FC-1 layer				
SVM	85.6	82.4	88.5	86.3
KNN	84.2	82.2	86.2	83.7
GB	87.1	83.0	88.5	87.2
RF	88.7	85.2	89.3	87.6
Analysis 2: PD prediction using extracted features from 3D-CNN FC-2 layer				
SVM	85.9	84.0	86.4	84.3
KNN	86.2	82.2	87.2	83.9
GB	90.1	86.0	92.0	88.9
RF	88.3	85.9	89.3	87.6
Analysis 3: PD prediction using feature fusion from FC-1 and FC-2 layer (i.e. FC-3)				
SVM	90.5	88.0	91.9	89.1
KNN	88.9	86.2	90.2	87.4
GB	93.5	91.0	96.2	92.5
RF	91.8	87.4	93.3	90.3
Analysis 4: PD prediction using 3DResNet features				
SVM	85.3	82.1	86.3	85.1
KNN	88.1	83.2	89.9	87.4
GB	90.0	88.3	92.5	91.0
RF	87.2	85.7	89.1	86.2
Analysis 5: PD prediction using EfficientNet features				
SVM	92.0	89.0	96.0	92.5
KNN	91.5	88.5	95.5	92.0
GB	92.8	90.0	97.0	93.5
RF	93.0	89.8	96.8	93.3
Analysis 6: PD prediction using Vision Transformer features				
SVM	91.5	89.5	96.5	93.0
KNN	92.5	89.0	96.0	92.5
GB	92.0	90.5	97.5	94.0
RF	91.2	90.2	97.2	93.7

### Model-2 (3D-ResNet)

The detailed model architecture of 3D-ResNet is as shown in Fig. 3. Features were extracted from a fully connected layer, FC-4, and then processed with a focus on classification using various machine learning models. The Gradient boosting-based ML algorithm achieved a maximum accuracy of 90%. The detailed performance analysis of the various ML models is elaborated in Table 4.

### Model development and optimization

The 3D CNN and 3D ResNet models were trained using MRI scans, with the 3D CNN employing pooling, activation and convolutional layers and the 3D ResNet using residual blocks. Data augmentation was applied to enhance robustness. Hyperparameters were optimized via grid search and cross-validation, using techniques like Adam and SGD with early stopping. Models were validated on a separate dataset to monitor overfitting and evaluated using metrics like accuracy, precision, recall, and F1-score. A lower learning rate in both models enhances model precision and stability, preventing overfitting and ensuring better convergence. A detailed comparison of both model training and hyperparameter tuning is comprehensively presented in Table 5.

**Table 5:** Comparison of training and validation details for 3D CNN and 3D ResNet Models.

Aspects	3D CNN	3D ResNet
Training data split	70% for Train and 15% for Test and validation	70% for Train and 15% for Test and validation
Learning rate	0.001	0.0001
Number of epochs	30	30
k-fold cross validation	5	5
Regularization (weight decay)	L2 Regularization (0.0001)	L2 Regularization (0.0001)
Optimization method	Grid search	Grid search
Validation performance	Selected the optimal hyperparameters based on best validation performance.	Selected the optimal hyperparameters based on best validation performance.

### Model-3 (without optimization)

In this approach, features were extracted from the layers of FC-3 of a 3D-CNN model and FC-4 of a 3D ResNet model. These extracted features from two different architectures were concatenated by using CCA. The fusion approach is carried out to integrate the complementary information obtained from two different models, thereby enhancing the classification accuracy. After the fusion process, the GB model with the highest accuracy is probed for performance enhancement with a 15-fold cross-validation technique, resulting in a further improved accuracy of 4.8%.

**Table 6:** Performance analysis of model-3 and model-4.

S.no	Cross validation	Accuracy	Recall	Precision	F1-Score
Model 3 (without optimization)					
1	5-fold	0.916	0.886	0.935	0.905
2	10-fold	0.924	0.882	0.928	0.918
3	15-fold	0.948	0.904	0.956	0.938
Model 4 (with optimization)					
1	5-fold	0.959	0.907	0.986	0.929
2	10-fold	0.962	0.893	0.988	0.934
3	15-fold	0.972	0.906	0.985	0.931

### Model-4 (with optimization)

The fusion approach carried out using CCA is cursed by higher dimensionality and sensitivity to outliers. There is a strong demand for the selection of optimal features with a novel optimization strategy. Hence, this study applies a bio-inspired optimization technique using WOA to select the optimal fused features. This optimization aims to refine the feature representation and thereby improve the model’s performance. With the optimized features, the GB algorithm, with a 15-fold cross-validation approach, results in an improved accuracy of 97.2%. This superior performance clearly indicates that the optimized features lead to more robust and generalizable models. The performance analysis of both Model 3 and Model 4 is presented in Table 6.

## Discussion

This section highlights the results obtained by using four different methodologies for the diagnosis of PD. Designed and built a Model-1 (3D CNN) architecture, refining it through several ablation studies. This network is capable of automatic feature extraction, demonstrating its effectiveness through performance metrics: 93.5% accuracy, 91.0% precision, 96.2% recall, and an overall F1-score of 92.5%. Previous studies^15^ have shown promising results in predicting the PD in the earlier stages, using the 3D improved ResNet architecture. The model-2 approach involves using an improved ResNet for automatic feature extraction, followed by classification using various ML algorithms. This model achieved 90% accuracy. While the performance of Model 2 is not as exceptional as that of Model 1, it is still effective, showing the capability to identify PD from T2-weighted images with satisfactory results.

To further improve the model performance, a based fusion technique is applied, which concatenates features from 3D-CNN and 3D-ResNet models. This approach blends the complementary information of both models to enhance performance. It is important to note that this fusion technique results in an improved accuracy of 2% compared to Model 1 performance. However, this integration may result in higher computational complexity and the issue of overfitting. To mitigate these issues, feature optimization methods were explored in this study. The proposed Model-4 is developed using bio-inspired WOA optimization techniques, and the performance is compared with four other optimization methods: Particle Swarm Optimization (PSO), Gravitational Search Algorithm (GSA), Genetic Algorithm (GA), and Ant Colony Optimization (ACO). These methods are selected for comparison based on their efficiency in addressing meta-heuristic optimization problems. The parameter values for each of these algorithms are provided in these algorithms are provided in Table 7.

**Table 7:** Parameter values for all algorithms.

Algorithms	Parameter	Value
PSO	Cognitive factor (c1)	2
	Social factor (c2)	2
	Inertia weight (w)	0.9
GSA	Gravitational constant (G0)	100
	Constant (alpha)	20
GA	Crossover rate (CR)	0.8
	Mutation rate (MR)	0.01
	Tournament size (Ts)	3
ACO	Pheromone value (tau)	1
	Heuristic desirability (eta)	1
	Control pheromone (alpha)	1
	Control heuristic (beta)	0.1
	Pheromone trail decay coefficient (rho)	0.2
WOA	Constant (b)	1
	Threshold (thres)	0.5
Search settings	Population Size	100
	Number of iterations	200
	Lower and upper bound	0 and 1
	No. of independent runs	20

**Figure 5:**
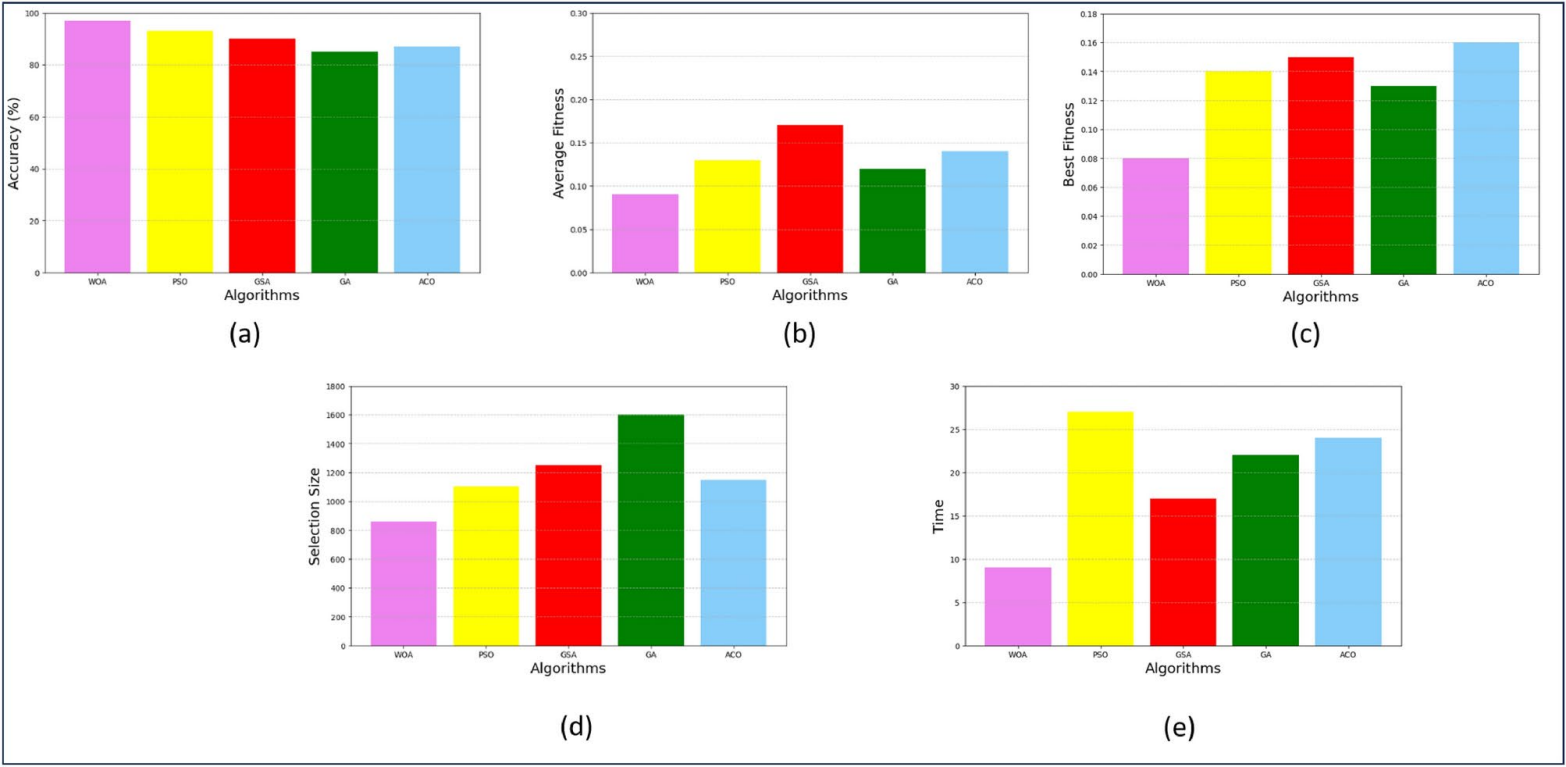
The overall performance comparison of algorithms across different metrics (**a**) accuracy, (**b**) average fitness, (**c**) best fitness, (**d**) selection size, (**e**) time.

The performance of these optimization algorithms is assessed based on several evaluation criteria, including accuracy, best fitness, average fitness, selection size, and time. The comparison of the algorithm’s performance across various metrics is illustrated in Fig. 5. The comparison clearly indicates that WOA demonstrates better accuracy compared to other optimization techniques. Other factors that determine the performance include the computational time complexity, the size of the selected optimized features and the value of the cost function. Based on this comparative analysis, it is concluded that the WOA algorithm exhibits better performance than other meta-heuristic algorithms.

**Figure 6:**
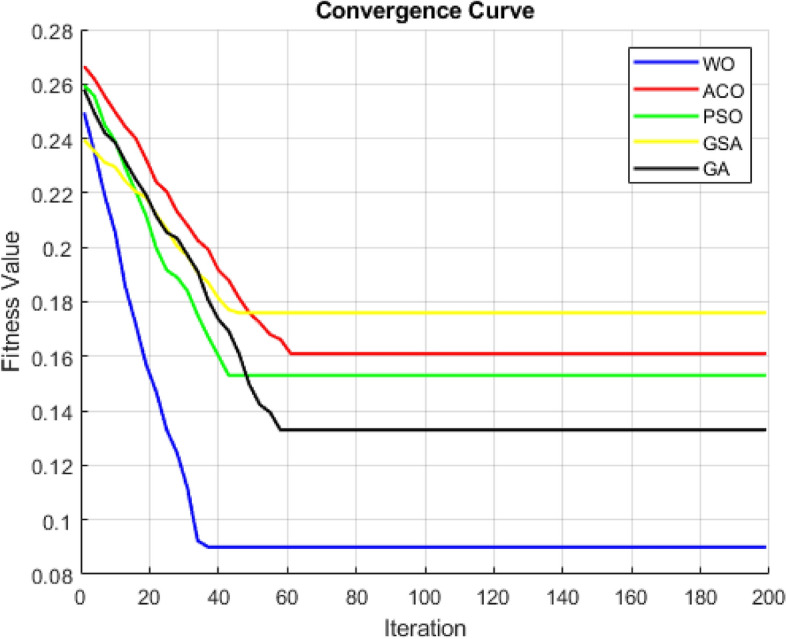
Convergence curves generated using various algorithms for the PPMI dataset.

Figure 6 display the convergence curves generated using different optimization algorithms for the PPMI dataset. These curves represent the fitness values obtained across 200 iterations for all meta-heuristic optimization algorithms. When compared, it is found that the WOA algorithm achieves the lowest fitness values compared to GA and PSO, indicating relatively better performance in terms of fitness.

**Figure 7:**
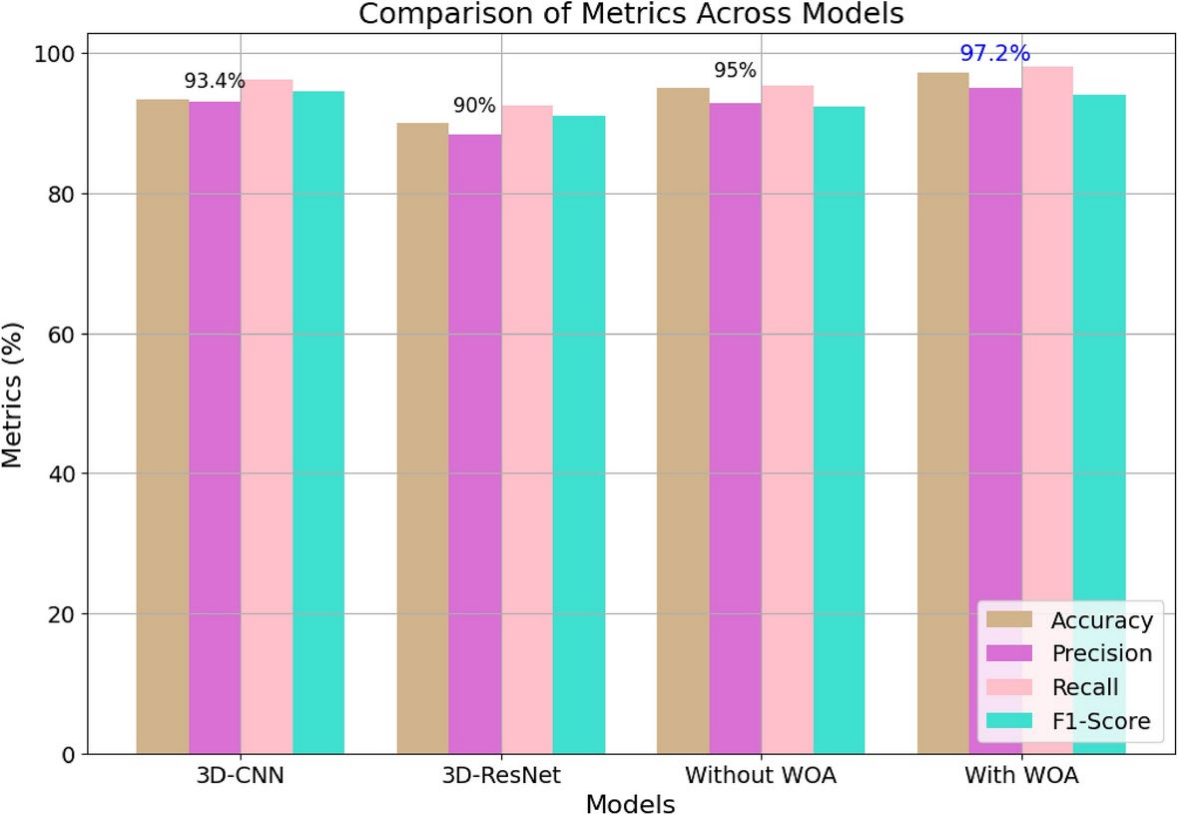
Comparison of performance metrics for the proposed method.

The proposed Model-4, using the WOA method, demonstrates a significant improvement of 5% compared to Model-1. This optimization step dramatically enhances the performance metrics, achieving impressive results with an accuracy of 97.2%, precision of 90.6%, recall of 98.5%, and F1-score of 92.1%. These findings highlight that substantial improvement in performance is achieved by deploying the optimal feature selection strategy. The comparison of various model performance metrics used in this study is illustrated in Fig. 7.

Table 8 presents a comparison between the proposed approach and the state-of-the-art methods in the literature. The comparison is performed with accuracy as a critical metric. The dataset used in each study is described in the third column, and the models used in this study, along with other state-of-the-art techniques, are detailed in column four.

**Table 8:** Comparing the suggested approach with earlier research.

References	Year	Dataset	Method	Accuracy (%)
^32^	2020	PPMI	Ensemble method	96.4
^33^	2024	Figshare and Brats	Residual fused Shepard CNN	94.0
^34^	2022	PPMI	Stacked Sparse Autoencoder + DL	93.3
^35^	2020	PPMI	ML	95.3
^36^	2021	PPMI	ML + DL	90.0
^37^	2023	PPMI	MNC-Net	95.5
Proposed framework				
3D-CNN	–	PPMI	3D-CNN	93.4
3D-ResNet	–	PPMI	3D-ResNet	97.2
Without optimization	–	PPMI	ML (gradient boosting)	90.0
With optimization	–	PPMI	ML (gradient boosting)	95.0

## Conclusion

In this paper, a novel method is proposed that begins with extracting features from two different deep neural network architectures, focusing on fusing this feature information using CCA. Optimal features are then selected from the fused features using bio-inspired optimization strategies, specifically WOA, resulting in an enhanced classification accuracy of 97.2%. Novel features were extracted from preprocessed MR images using two models: an improved ResNet and a specially designed 3D-CNN architecture, following several ablation studies. The classification accuracy achieved by using ResNet and the proposed 3D-CNN architecture was 90% and 93.4%, respectively. Incorporating feature fusion using CCA and whale optimization-based optimal feature selection led to a 2% improvement in accuracy. The optimal feature selection was compared with metaheuristic algorithms, including WOA, PSO, GA, GSO, and ACO. The comparative study, based on the convergence diagram, found that WOA produced better results than state-of-the-art methods in the literature. A slight difference in model accuracy, approximately 1-1.3%, was observed between 1.5T and 3T MRI scans. However, the proposed framework maintained high accuracy and generalizability, demonstrating robustness across varying image qualities.

The proposed framework offers several advantages, including high accuracy and generalizability, making it robust across different MRI image qualities. However, challenges such as data privacy and device interoperability must be addressed to ensure effective implementation. The current model is trained and tested with PPMI data, which is open for research, whereas, while implementing this framework in real-time and continuously updated with real-time data, informed consent is obtained from all patients, with clear communication that only selected MRI features will be used for research, ensuring transparency and privacy. Additionally, adopting our model may require investment in local servers, while future work could focus on utilizing cloud-based solutions to streamline data handling and eliminate the need for local infrastructure. Training should emphasize identifying and excluding failed scans, such as those affected by electronic noise, artifacts, or patient movement, before data input. The framework enhances patient privacy by storing only essential features extracted from MRI images using WOA, avoiding the storage of original images or raw data to minimize unauthorized access and align with regulations.

In future research, the focus will be on refining the optimization algorithm, implementing multi-objective optimization, and enhancing model interpretability. The proposed framework can be extended to diagnose other neurodegenerative diseases, such as Alzheimer’s and Huntington’s, which require additional features along with the features extracted in this work, like amyloid and tau imaging, to identify their presence accurately. By incorporating datasets like the Alzheimer’s Disease Neuroimaging Initiative (ADNI) and adjusting our framework to include these specific imaging features, we can enhance disease-specific sensitivity. Furthermore, the integration of IoT and AI technologies will be explored to enhance healthcare applications for PD. This includes utilizing wearable sensors for continuous monitoring and employing AI algorithms for data analysis to improve diagnostics and personalized care.

## Data Availability

The training and testing data used in this study is available from the Parkinson’s Progression Markers Initiative (PPMI) which is an open-access database.
